# Prognostic significance of TCF21 mRNA expression in patients with lung adenocarcinoma

**DOI:** 10.1038/s41598-017-02290-2

**Published:** 2017-05-17

**Authors:** Jian Xiao, Aibin Liu, Xiaoxiao Lu, Xi Chen, Wei Li, Shuya He, Bixiu He, Qiong Chen

**Affiliations:** 1 Department of Geriatrics, Respiratory Medicine, Xiangya Hospital of Central South University, Changsha, China; 2 Department of Geriatrics, Xiangya Hospital of Central South University, Changsha, China; 3 Department of Respiratory Medicine, Xiangya Hospital of Central South University, Changsha, China; 4 Department of Geriatrics, Clinical Laboratory, Xiangya Hospital of Central South University, Changsha, China; 5 Department of Biochemistry & Biology, University of South China, Hengyang, China

## Abstract

Several prognostic indicators have shown inconsistencies in patients of different genders with lung adenocarcinoma, indicating that these variations may be due to the different genetic background of males and females with lung adenocarcinoma. In this study, we first used the Gene-Cloud of Biotechnology Information (GCBI) bioinformatics platform to identify differentially expressed genes (DEGs) that eliminated gender differences between lung adenocarcinoma and normal lung tissues. Then, we screened out that transcription factor 21 (TCF21) is a hub gene among these DEGs by creating a gene co-expression network on the GCBI platform. Furthermore, we used the comprehensive survival analysis platforms Kaplan-Meier plotter and PrognoScan to assess the prognostic value of TCF21 expression in lung adenocarcinoma patients. Finally, we concluded that decreased mRNA expression of TCF21 is a predictor for poor prognosis in patients with lung adenocarcinoma.

## Introduction

Lung cancer is a malignant tumor characterized by uncontrolled cell growth in tissues of the lung and bronchus. Except for prostate cancer in males and breast cancer in females, lung cancer is the second most commonly diagnosed cancer in men and women^1^. It is the most common cause of cancer death in humans and accounts for more than one-quarter of all cancer deaths^1^. Moreover, the overall age-adjusted 5-year relative survival for lung cancer is 15.5% in males and 20.3% in females^2^. Therefore, further researches are needed to develop treatments for lung cancer, including the identification of prognostic factors for lung cancer patients.

Non-small cell lung cancer (NSCLC) accounts for 85% of total lung cancer cases^3^. According to the latest statistics from the SEER Cancer Statistics Review, from 2006 to 2012, the average 5-year relative survival rate of NSCLC in males is 19.2% and in females is 26.8%^4^. The major histological phenotypes of NSCLC are classified as adenocarcinoma, squamous cell carcinoma and large cell carcinoma. However, lung adenocarcinoma (approximately 50% of cases) is the most important pathological type of NSCLC^5^. In recent years, many prognostic factors for the survival of lung adenocarcinoma patients have been reported by researchers^6–12^. However, some of these indicators have inconsistent prognostic value in patients with lung adenocarcinoma according to different gender^13–17^. We hypothesized that one of the causes of this inconsistency is due to the different genetic background of lung adenocarcinoma in males and females.

In this study, we used the Gene-Cloud of Biotechnology Information (GCBI) bioinformatics platform to identify differentially expressed genes (DEGs) that eliminated gender differences between lung adenocarcinoma and normal lung tissues. Among these DEGs, we found that transcription factor 21 (TCF21) is a hub gene. Furthermore, using the comprehensive survival analysis platforms Kaplan-Meier plotter and PrognoScan, we showed that the decreased mRNA expression of TCF21 is an unfavorable prognostic factor for lung adenocarcinoma patients.

## Results

### Major sample characteristics

We used two Gene Expression Omnibus (GEO) datasets (GSE40791^18^ and GSE10072^19^) in this study. Both of these datasets were included in the GCBI bioinformatics analysis platform. GSE40791 included 94 lung adenocarcinoma frozen tissues (53 derived from males, 41 derived from females) and 100 non-neoplastic lung samples (58 derived from males, 42 derived from females) (Supplementary Table S1). GSE10072 included 58 tumor samples (35 derived from males, 23 derived from females) and 49 non-tumor tissues (34 derived from males, 15 derived from females) (Supplementary Table S2).

### Research design

**Figure 1:**
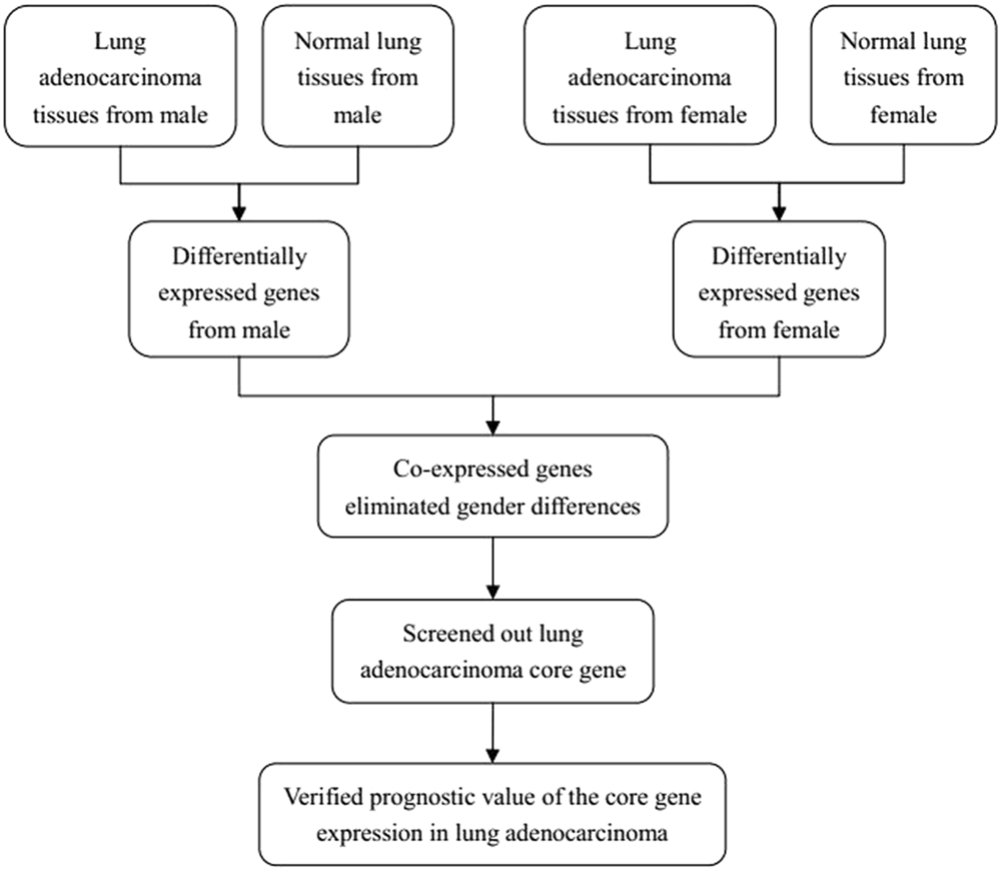
Flow diagram of the study design.

In our study, we wanted to identify the DEGs that eliminated gender differences between lung adenocarcinoma and normal lung tissues. Therefore, we first divided both lung adenocarcinoma and non-neoplastic lung samples into two groups according to gender. Using the GCBI bioinformatics analysis platform, we then obtained two DEG datasets from males and females. Then, by selecting the intersection of these two datasets, we obtained the co-expressed DEGs that eliminated gender differences. Next, we created a gene co-expression network to screen out hub genes in the co-expressed DEGs. Finally, we verified the prognostic value of core gene expression for patients with lung adenocarcinoma using the comprehensive survival analysis platforms Kaplan-Meier plotter and PrognoScan. The flow diagram of our study design is shown in Fig. 1.

### TCF21 is the core gene in the DEGs obtained from GSE40791

**Figure 2:**
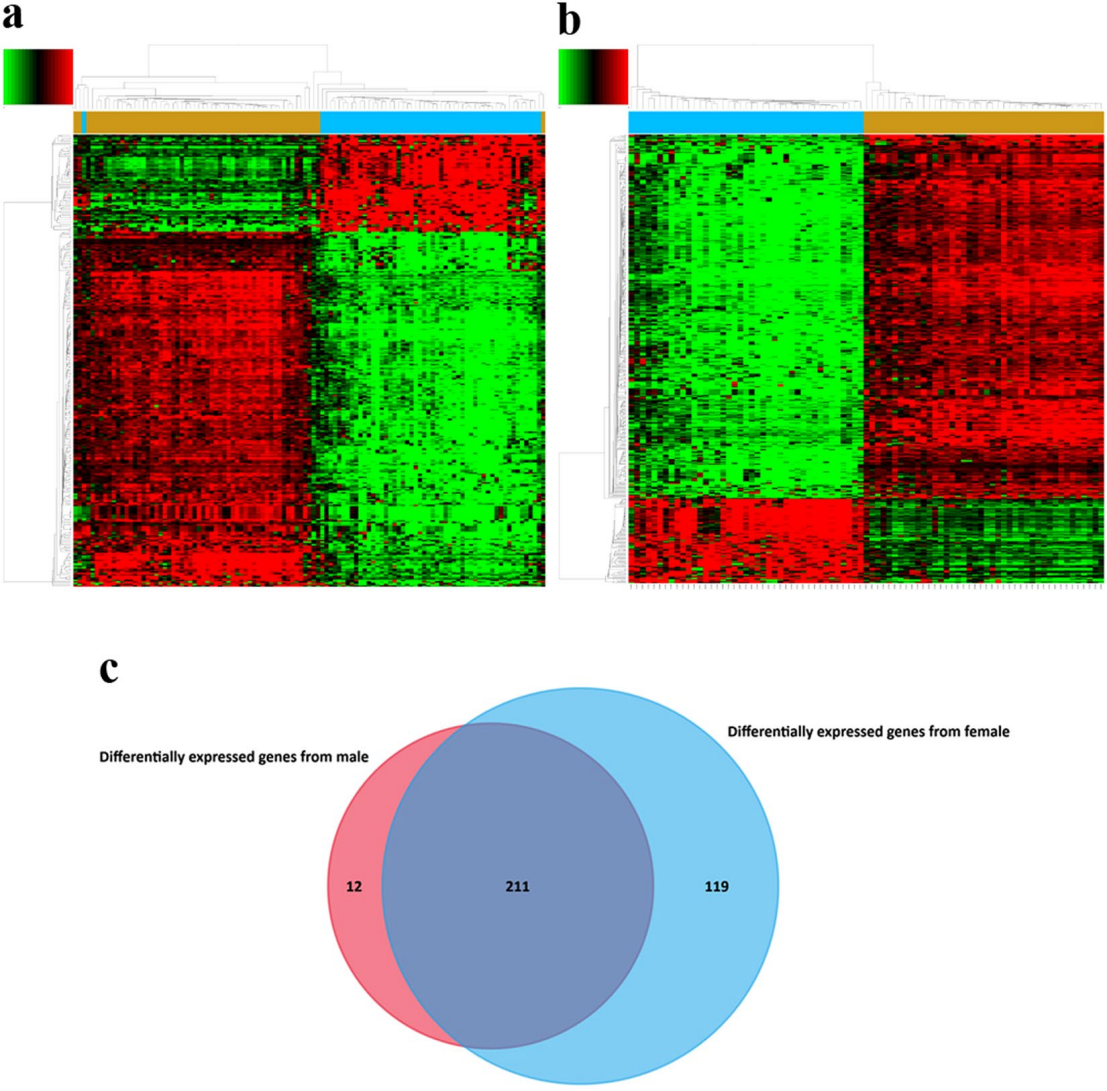
DEGs between lung adenocarcinoma and normal lung tissues for GSE40791. (**a**) Heat map for potential DEGs (n = 321) from males (lung adenocarcinoma n = 53, in blue; normal lung tissues n = 58, in yellow). (**b**) Heat map for potential DEGs (n = 463) from females (lung adenocarcinoma n = 41, in blue; normal lung tissues n = 42, in yellow). (**c**) Duplicate expressions with the same gene symbol and those without a specific gene symbol were removed; the Venn diagram shows DEGs (n = 223) from males, DEGs (n = 330) from females and the co-expressed genes (n = 211) that eliminated gender differences.

**Figure 3:**
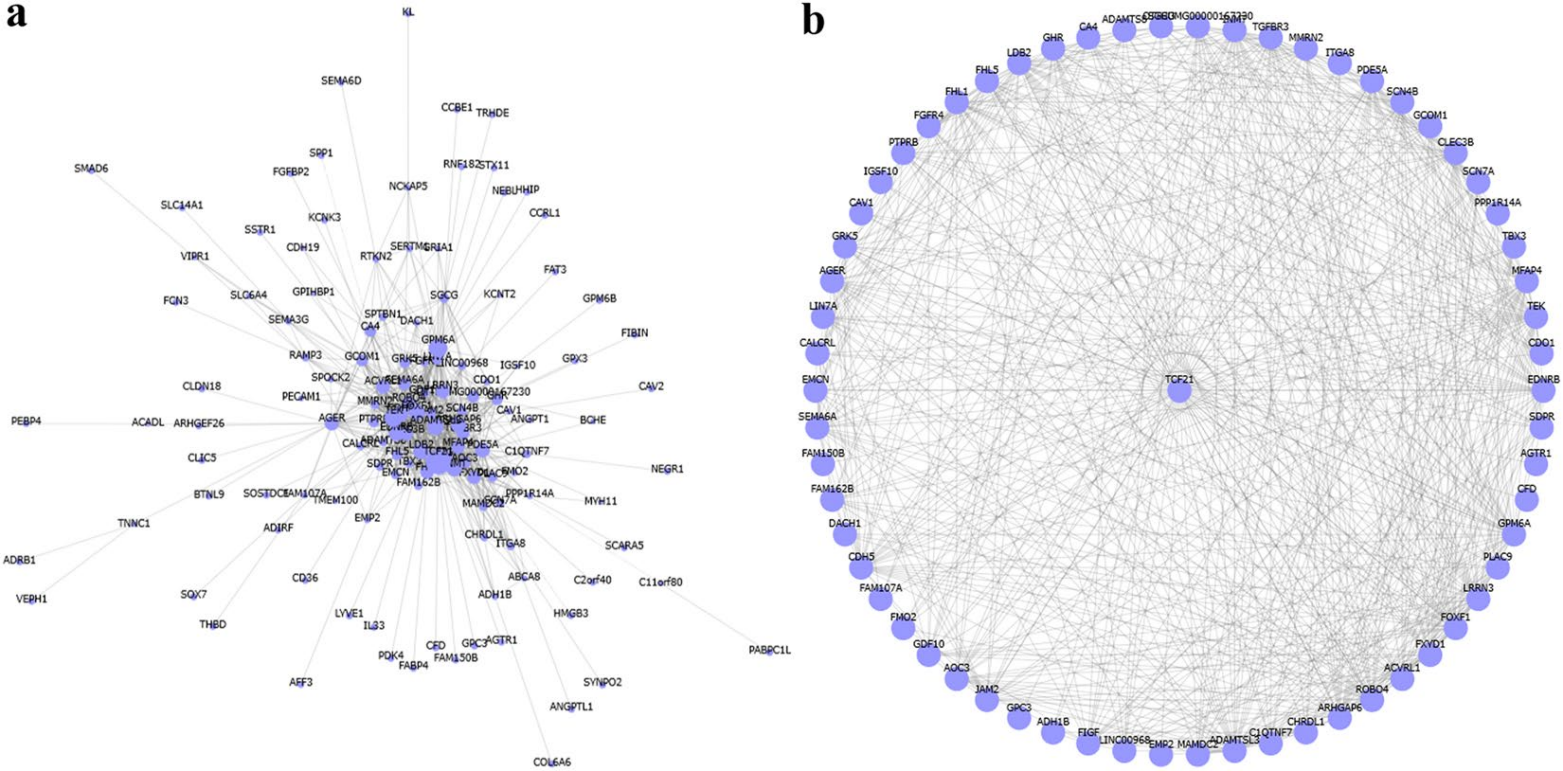
Gene co-expression network of the DEGs that eliminated gender differences for GSE40791. (**a**) The gene co-expression network. (**b**) Sub-graph shows that TCF21 has the most gene connections in the gene co-expression network.

We identified 321 potential DEGs from males (Fig. 2a and Supplementary Table S3) and 463 potential DEGs from females (Fig. 2b and Supplementary Table S4). To remove duplicate expressions from the same gene and expressions from unannotated genes, 223 DEGs (Supplementary Table S5) and 330 DEGs (Supplementary Table S6) were selected from the male and female groups. By taking the intersection of these two DEGs datasets, 211 co-expressed DEGs that eliminated gender differences were found (Fig. 2c). Among them, 131 DEGs were incorporated into the gene co-expression network (Fig. 3a). As shown in Table 1 and Fig. 3b, we found that TCF21 had the most gene connections in the gene co-expression network. Therefore, we hypothesized that TCF21 is the core gene in the DEGs between lung adenocarcinoma and normal lung tissues.

**Table 1:** Top 10 hub genes in the gene co-expression network of the DEGs that eliminated gender differences for GSE40791.

Gene Symbol	Degree	Description
TCF21	62	transcription factor 21
GPM6A	49	glycoprotein M6A
TEK	46	TEK tyrosine kinase, endothelial
ADAMTSL3	40	ADAMTS-like 3
FOXF1	39	forkhead box F1
CDH5	38	cadherin 5, type 2 (vascular endothelium)
LDB2	38	LIM domain binding 2
EDNRB	37	endothelin receptor type B
ARHGAP6	36	Rho GTPase activating protein 6
ROBO4	35	roundabout, axon guidance receptor, homolog 4 (Drosophila)

### TCF21 is also the core gene in the DEGs obtained from GSE10072

**Figure 4:**
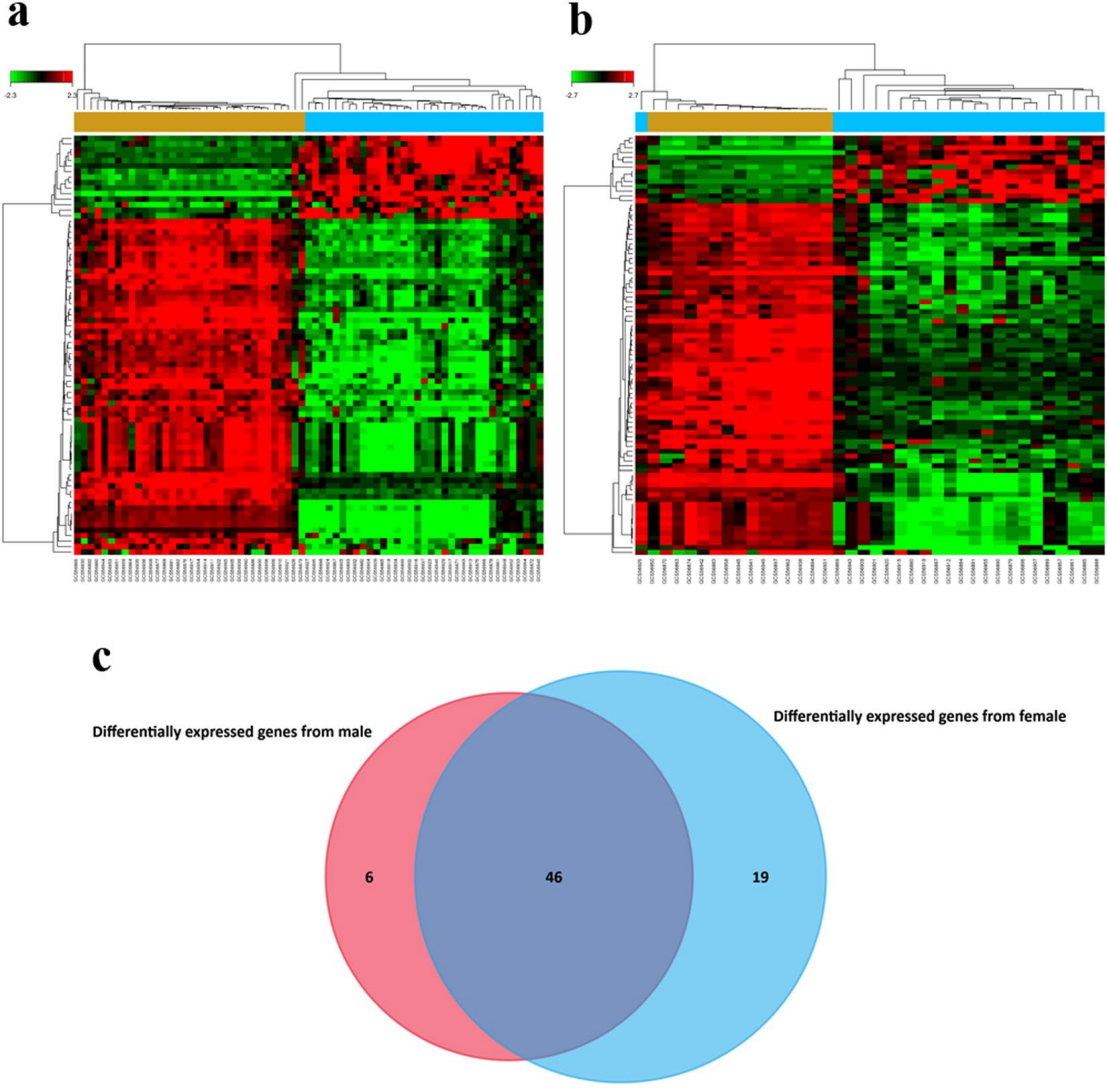
DEGs between lung adenocarcinoma and normal lung tissues for GSE10072. (**a**) Heat map for potential DEGs (n = 76) from males (lung adenocarcinoma n = 35, in blue; normal lung tissues n = 34, in yellow). (**b**) Heat map for potential DEGs (n = 87) from females (lung adenocarcinoma n = 23, in blue; normal lung tissues n = 15, in yellow). (**c**) Duplicate expressions with the same gene symbol and those without a specific gene symbol were removed; the Venn diagram shows DEGs (n = 52) from males, DEGs (n = 65) from females and the co-expressed genes (n = 46) that eliminated gender differences.

**Figure 5:**
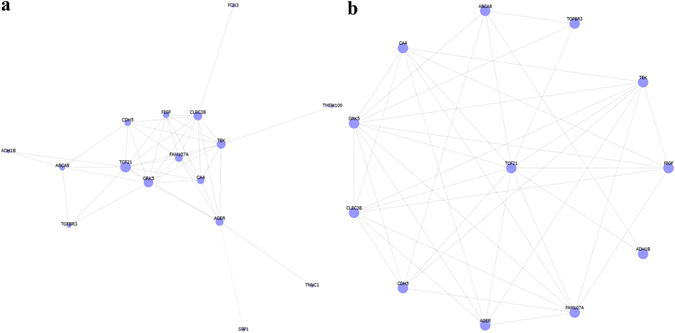
Gene co-expression network of the DEGs that eliminated gender differences for GSE10072. (**a**) The gene co-expression network. (**b**) The sub-graph shows that TCF21 has the most gene connections in the gene co-expression network.

To verify the results obtained from GSE40791, we then used the same process in GCBI to study the DEGs between lung adenocarcinoma and normal lung tissues from GSE10072. We identified 76 potential DEGs from males (Fig. 4a and Supplementary Table S7) and 87 potential DEGs from females (Fig. 4b and Supplementary Table S8). To remove duplicate expressions from the same gene and expressions from unannotated genes, 52 DEGs (Supplementary Table S9) and 65 DEGs (Supplementary Table S10) were selected from the male and female groups, respectively. By taking the intersection of these two DEGs datasets, 46 co-expressed DEGs that eliminated gender differences were found (Fig. 4c). Among them, 16 DEGs were incorporated into the gene co-expression network (Fig. 5a). As shown in Table 2 and Fig. 5b, we again found that TCF21 has the most gene connections in the gene co-expression network. Consequently, we confirmed that TCF21 is the core gene in the DEGs between lung adenocarcinoma and normal lung tissues.

**Table 2:** Top 10 hub genes in the gene co-expression network of the DEGs that eliminated gender differences for GSE10072.

Gene Symbol	Degree	Description
TCF21	11	transcription factor 21
GRK5	10	G protein-coupled receptor kinase 5
CLEC3B	9	C-type lectin domain family 3, member B
TEK	9	TEK tyrosine kinase, endothelial
FAM107A	8	family with sequence similarity 107, member A
AGER	8	advanced glycosylation end product-specific receptor
CA4	7	carbonic anhydrase IV
FIGF	6	c-fos induced growth factor (vascular endothelial growth factor D)
CDH5	6	cadherin 5, type 2 (vascular endothelium)
ABCA8	5	ATP-binding cassette, sub-family A (ABC1), member 8

### Decreased mRNA expression of TCF21 is an unfavorable prognostic factor for lung adenocarcinoma

**Figure 6:**
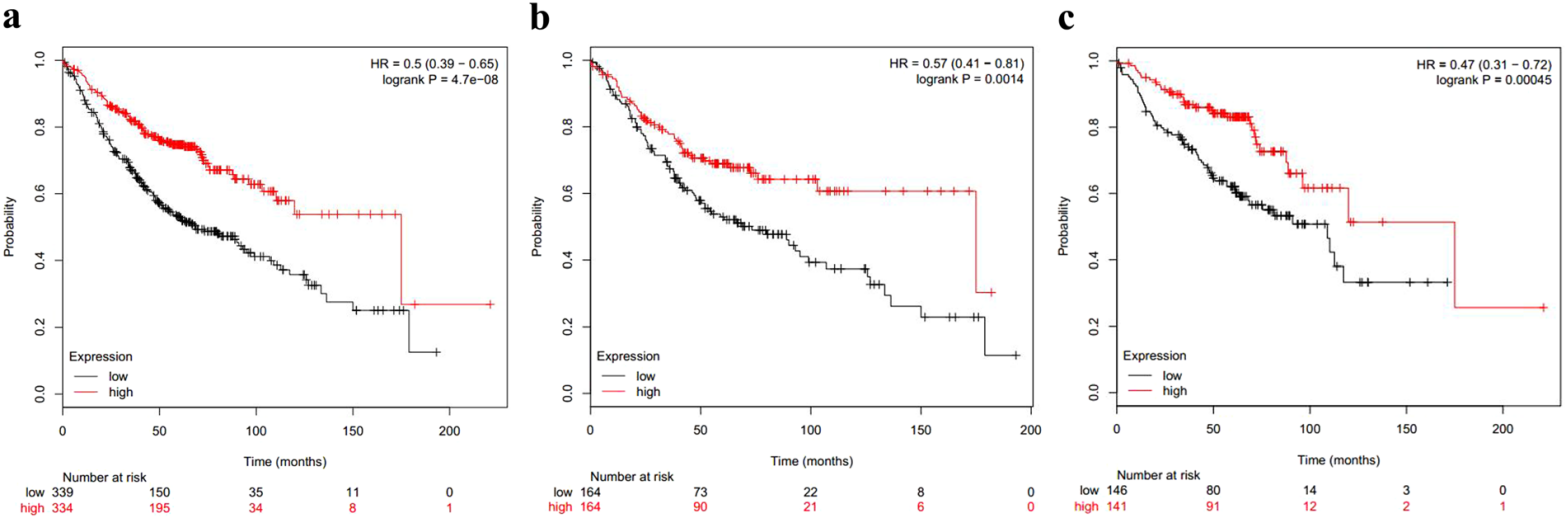
Kaplan–Meier survival curves generated from Kaplan-Meier plotter for TCF21 mRNA expression in patients with lung adenocarcinoma. Overall survival curves for lung adenocarcinoma patients that ignored gender differences (**a**), in male patients (**b**) and in female patients (**c**). HR = hazard ratio.

**Figure 7:**
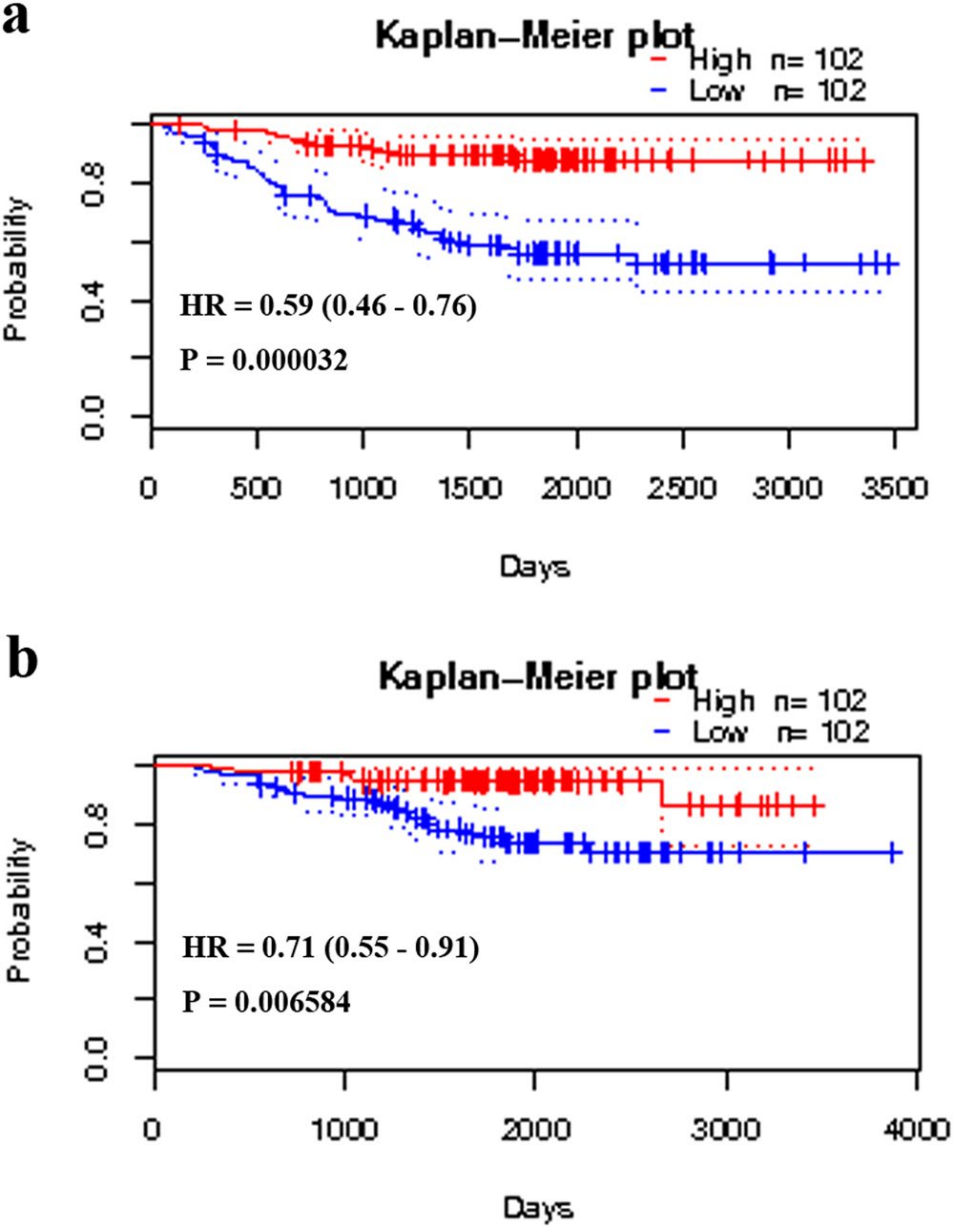
Kaplan–Meier survival curves generated from PrognoScan for TCF21 mRNA expression in patients with lung adenocarcinoma. Relapse-free survival curves (**a**) or overall survival curves (**b**) for lung adenocarcinoma patients in the GEO dataset GSE31210. HR = hazard ratio.

Compared with non-neoplastic lung tissues, TCF21 was decreased in the lung adenocarcinoma samples (Supplementary Tables S3–S10). Furthermore, TCF21 is the core gene in the DEGs between lung adenocarcinoma and normal lung tissues (Figs 3 and 5). Accordingly, we hypothesized that TCF21 expression may be a prognostic factor for patients with lung adenocarcinoma. Using the comprehensive Kaplan-Meier survival analysis platform, we discovered that decreased mRNA expression of TCF21 is an unfavorable prognostic factor of overall survival for patients with lung adenocarcinoma (hazard ratio, HR = 0.50; 95% confidence interval, 95% CI: 0.39–0.65; P = 4.7e^−08^; n = 673) (Fig. 6a). When further considering the gender difference, decreased mRNA expression of TCF21 has similar prognostic value in both males (HR = 0.57; 95% CI: 0.41–0.81; P = 0.0014; n = 328) and females (HR = 0.47; 95% CI: 0.31–0.72; P = 0.00045; n = 287) (Fig. 6b and c). Simultaneously, results generated from PrognoScan based on the GEO dataset of GSE31210 indicated that decreased mRNA expression of TCF21 is not only an unfavorable prognostic factor for lung adenocarcinoma patients’ relapse-free survival (HR = 0.59; 95% CI: 0.46–0.76; P = 0.000032; n = 204) (Fig. 7a) but also for overall survival (HR = 0.71; 95% CI: 0.55–0.91; P = 0.006584; n = 204) (Fig. 7b). However, also using the comprehensive survival analysis platforms Kaplan-Meier plotter and PrognoScan, we found that TCF21 expression has no prognostic value in patients with lung squamous cell carcinoma (data not shown).

## Discussion

To assess the prognostic value of gene expression for lung cancer patient survival, the expression of the genes was normally detected by immunohistochemistry in protein level^20–22^. However, the expression level of mRNA detected by polymerase chain reaction also performed reliable prognostic value for lung cancer patients^23, 24^. In this study, we extracted gene expression array data from GEO datasets using the GCBI bioinformatics analysis platform and further assessed these data using the comprehensive survival analysis platform Kaplan-Meier plotter and PrognoScan. We finally found that decreased TCF21 mRNA expression is an unfavorable prognostic factor for patients with lung adenocarcinoma. Our study indicated that comprehensive utilization of bioinformatics analyses is a good strategy to study prognosis of lung adenocarcinoma and may be useful to assess prognosis for other cancer types.

Previous studies did not consider the effect of gender differences in DEGs between lung and normal tissues at the design stage. Because of the different genetic background between males and females, we considered that lung adenocarcinomas with different gender origins may also have significant genetic difference. In our study, to eliminate the impact of gender differences, we divided both lung adenocarcinoma and non-neoplastic lung samples into two groups according to gender. Using comprehensive bioinformatics analyses, we reported that the decreased mRNA expression of TCF21 is an unfavorable prognostic factor for patients with lung adenocarcinoma. To our knowledge, this is the first study to investigate the prognostic value of TCF21 in patients with this specific lung cancer type.

TCF21 (also known as Capsulin, Pod-1 or Epicardin) is a member of the basic-helix-loop-helix transcription factor family^25–27^. During embryogenesis, TCF21 is crucial for the development of a number of cell types in the spleen^28^, heart^29^, kidney^30^ and lung^30^. However, TCF21 is also important in cancer development. *In vitro*, it can affect tumor cell cycle balance^31^ and suppress cancer cell proliferation, migration^32^ and invasion^32, 33^. Additionally, mouse model experiments showed that TCF21 can significantly reduce tumor growth *in vivo* ^34, 35^. Consequently, TCF21 is considered to be a potential tumor suppressor^34, 36^.

Unfortunately, TCF21 expression is commonly deregulated by promoter hypermethylation in different types of cancer^32, 33, 37–40^. This phenomenon is detrimental for cancer patients. Study reported that TCF21 promoter methylation is correlated with decreased survival in patients with metastatic skin melanoma^33^. Low expression of TCF21 is an independent prognostic factor for poor survival in patients with clear cell renal cell carcinoma^41^ or gastric cancer^42^.

For lung cancer, previous studies demonstrated that aberrant TCF21 promoter methylation existed in 9 of 10 lung cancer cell lines^39^ and the majority of lung cancer tissues (70–81%)^34, 38, 39^. Richards KL and colleagues^38^ reported that 84% of non-small cell lung cancer samples showed decreased TCF21 protein expression. TCF21 expression was not correlated with gender and was lower in adenocarcinoma than in squamous cell carcinoma^38^, indicating that TCF21 is a crucial tumor suppressor for lung adenocarcinoma in both males and females. We corroborated these findings in the present study. Recently, Wu H and colleagues^35^ found that low TCF21 expression is associated with poor survival for lung cancer using the survival analysis platform Kaplan-Meier plotter. In our study, we comprehensively used both Kaplan-Meier plotter and PrognoScan to first conclude that decreased mRNA expression of TCF21 is an unfavorable prognostic factor in lung adenocarcinoma patients without gender difference and that TCF21 expression showed no prognostic value in patients with lung squamous cell carcinoma. We consider that maybe TCF21 is a specific prognostic factor in lung adenocarcinoma rather than in lung squamous cell carcinoma, for which this point has not taken into account in previous studies.

We know that TCF21 is a crucial tumor suppressor, and TCF21 expression is commonly deregulated by aberrant promoter methylation in different types of cancer. Additionally, decreased expression of TCF21 is a poor prognostic factor for cancer patients. However, we know little about the tumor suppressor mechanism of TCF21. Therefore, in the future, more studies should be conducted to elucidate the mechanisms of TCF21 tumor suppressor function. In addition, the bioinformatics analysis platforms of GCBI, Kaplan-Meier plotter and PrognoScan used in this study were based on the method of unsupervised analysis of gene expression profiles (named the “one-step-clustering” approach by Li J *et al*.^43^). Because of the extremely high variability in gene expression profiles between individual tumors and because “passenger signals” may mask the “real” cancer gene signals, our results derived from this “one-step-clustering” approach may be less robust and accurate^43, 44^.

In summary, we first used the GCBI bioinformatics analysis platform to identify DEGs that eliminated gender differences between lung adenocarcinoma and normal lung tissues, which showed that TCF21 is the hub gene. Then, using the comprehensive survival analysis platforms of Kaplan-Meier plotter and PrognoScan, we concluded that decreased mRNA expression of TCF21 is a poor prognostic factor in patients with lung adenocarcinoma.

## Methods

### Gene-Cloud of Biotechnology Information (GCBI)

GCBI (Shanghai, China, https://www.gcbi.com.cn) is a platform that combines a variety of research findings, genetic information, sample information, data algorithms and bioinformatics to create a “gene knowledge base,” which encompasses biology, medicine, informatics, computer science, mathematics, graphics and other disciplines. GCBI includes more than 120 million copies of genomic samples, approximately 90,000 copies of tumor samples and more than 17 million copies of genetic information. Therefore, GCBI is a good bioinformatics analysis platform and has provided data analysis support for many studies on cancer research^45–49^. In this study, we used GCBI to identify DEGs between lung adenocarcinoma and normal lung tissues and finally screened out that TCF21 is the hub gene in the DEG co-expression network. In the Differential Gene Expression Analysis module on the GCBI platform (Supplementary File), we chose a fold expression change >5 at cutoff values Q < 0.05 and P < 0.05 to screen out DEGs. Then, we selected the Gene Co-expression Network module on the GCBI platform to create a gene co-expression network for the DEGs that eliminated gender differences.

### Gene Expression Omnibus (GEO) DataSets

GEO DataSets (https://www.ncbi.nlm.nih.gov/gds) is the public repository for storing high throughput gene expression datasets at National Center of Biotechnology Information. In this study, we selected datasets according to the following inclusion criteria: (1) Human lung cancer specimens containing the pathological type of adenocarcinoma; (2) Normal lung tissues used as the controls; (3) Specimens had gender information; (4) Number of samples no less than 100; (5) Expression profiling by array and raw data had the CEL format. First, we used the search terms “Lung cancer [MeSH Terms] AND Homo sapiens [Organism] AND Adenocarcinoma [Description] CEL AND [Supplementary Files] AND Expression profiling by array [DataSet Type]” in the GEO DataSets to identify potential datasets. Then, we further screened these datasets according to the above inclusion criteria. Finally, two GEO datasets, GSE40791 and GSE10072, were included into our study.

### Kaplan-Meier plotter

The Kaplan-Meier plotter (http://kmplot.com/analysis/) is a comprehensive online platform that can assess the effect of 54,675 genes on survival based on 10,293 cancer samples. These include 2,437 lung cancer patients with a mean follow-up of 49 months. With a meta-analysis of published microarray datasets, this online tool is suitable for in silico validation of new biomarkers related to survival for patients with non-small cell lung cancer^50^. We used the Kaplan-Meier plotter to assess the prognostic value of TCF21 expression in patients with lung adenocarcinoma or squamous cell carcinoma.

### PrognoScan

PrognoScan (http://www.prognoscan.org/) is a comprehensive platform for evaluating potential tumor biomarkers and therapeutic targets. Based on a large collection of cancer microarray datasets with clinical annotation, PrognoScan is a useful online tool for assessing the association between specific gene expression and prognosis in patients with cancer^51^. We used the PrognoScan platform to validate the prognostic value of TCF21 expression in patients with lung adenocarcinoma or squamous cell carcinoma.

## Electronic supplementary material

Supplementary Table S1

Supplementary Table S2

Supplementary Table S3

Supplementary Table S4

Supplementary Table S5

Supplementary Table S6

Supplementary Table S7

Supplementary Table S8

Supplementary Table S9

Supplementary Table S10

Supplementary File
