# Supplementary material for: Prognostic significance of TCF21 mRNA expression in patients with lung adenocarcinoma
Source: Sci Rep. 2017 May 17;7:2027. doi: 10.1038/s41598-017-02290-2 (PMC5435710; doi:10.1038/s41598-017-02290-2)
Supplement: Supplementary file 11 — Supplementary File [file 41598_2017_2290_MOESM11_ESM.pdf]

Prognostic significance of TCF21 mRNA expression in patients with lung adenocarcinoma

Jian Xiao[1], Aibin Liu[2], Xiaoxiao Lu[1], Xi Chen[3], Wei Li[4], Shuya He[5], Bixiu He[1], and Qiong Chen[1]

[1]Department of Geriatrics, Respiratory Medicine, Xiangya Hospital of Central South University, Changsha, China
[2]Department of Geriatrics, Xiangya Hospital of Central South University, Changsha, China
[3]Department of Respiratory Medicine, Xiangya Hospital of Central South University, Changsha, China
[4]Department of Geriatrics, Clinical Laboratory, Xiangya Hospital of Central South University, Changsha, China
[5]Department of Biochemistry & Biology, University of South China, Hengyang, China

Correspondence and requests for materials should be addressed to Q. C. (E-mail: qiongch@163.com)

Method Description of Difference Analysis

There are mainly two partial contents in this manual:
Part one, the details of the difference analysis process;
Part two, the default parameters of the clustering heatmap.
Besides, the ranking method for the order differently significant genes are listed in the appendix.
In order to get high confidence analysis results with verifiability, GCBI recommend the number of each groups no less than 3. It means that you should have large samples to analyze your expression profile (use Method 2 or 3 for analysis). Moreover, for a reliable result used in the cross analysis (e.g. the joint analysis of miRNA and mRNA, the union or for multiple different results), GCBI recommend the number of samples for each platform no less than 10.
Part One: the details for screening the different genes
Step 1: the preprocess of the chip signal and filtering low variant genes
First, we pad the signal values for low abundance genes. That is to say the signal value low than log3 will be filled with log3. Besides, the no variant genes (same expression value) and low variant genes should be filtered. GCBI will filter these genes whose detected percentage below 50% in the whole expression profile.
Step 2: The analysis process of difference analysis for the expression profile
Method one, the FC method for the type of less than 3.
The format for as follows:

$$Fold\ Change = \begin{cases} \frac{2^{\bar{x}_1}}{2^{\bar{x}_2}} & if \quad \bar{x}_1 \geq \bar{x}_2 \\ -\frac{2^{\bar{x}_2}}{2^{\bar{x}_1}} & if \quad \bar{x}_1 < \bar{x}_2 \end{cases}$$

Where $\bar{x}_1$ and $\bar{x}_2$ was the mean of the gene signals in different conditions (experiment groups) respectively.
Method two, the SAM method for small samples, which the number of samples in each group no less than 3. We implement a series of steps to obtain the estimation of significance of difference and false discovery rate for every filtered gene:

(1) Calculate the exchange factor s_0.

Firstly, calculate the standard deviation for all genes s_i, denote s^α as the α percentile for s_i.

For the percentile value $q_1 < q_2 < \cdots < q_{100}$ of the s_i, calculate the statistic

$$v_j = mad\{d_i^\alpha = r_i/(s_i + s^\alpha)\,|s_i = [q_j, q_{j+1})\},$$

where mad denotes the mean absolute deviation. At last, we select the α (denote as $\hat{\alpha}$) to make the CV (coefficient of variation) of the v_j achieve minimum. Now, we get the exchange factor $s_0 = s^{\hat{\alpha}}$.

(2) Calculate the statistic value (d Score) for every gene

$$d_i = r_i/(s_i + s_0),$$

Where r_i reflects the difference in average level among different groups, s_i reflects the variation of sample population. See details in references [1, 2].

(3) Calculate the order statistic

$$d_{(1)} \leq d_{(2)} \leq \cdots \leq d_{(i)} \leq \cdots \leq d_{(p)}.$$

(4) In order to get the above statistic's estimate, we make a permutation method (In fact, a loop strategy through every sample, the total number no less than 1000. we omit the details) the expected distribution of d Score. We denote the estimated statistic values:

$$d^*_{(1)} \leq d^*_{(2)} \leq \cdots \leq d^*_{(i)} \leq \cdots \leq d^*_{(p)}.$$

(5) We obtain the order statistic value under the permutation:

$$\bar{d}_{(i)} = \frac{\sum_{i=1}^{1000} d^*_{(i)}}{1000}.$$

(6) By calculate the maximum distance between the order statistic $d_{(i)}$ and the expected order statistic $\bar{d}_{(i)}$, we would construct a series of reject region for q-value. In fact, we obtain a grid of delta values by dividing 50 equant delta value for the above distance.

(7) For a fixed delta value, by computing the difference $\Delta_{(i)} = d_{(i)} - \bar{d}_{(i)}$, we would find the nearest $\Delta_{(i)}$ for gene i. We mark the cut_up as $\min\{\Delta_{(i)} \geq \text{delta}\}$ for positive gene and the cut_down $\max\{\Delta_{(i)} \leq -\text{delta}\}$ as for negative gene. Then we call these genes significant positive gene whose difference large than cut_up value (we denote the number of these genes as $R_{(p)}$) and these genes significant negative gene whose difference small than cut_down value.

(8) Under the value of cut_up and cut_down, we simulate the operation of step (7) for the statistic obtained by step (4). Then we get the number of positive genes for mock random state (i.e. no less than 1000 permutation). Now, we would get the estimation of false discovery rate FDR (i.e. we mark the median of the number of false positive genes for the above permutation as $R_{(p)}$, then we get the positive $FDR = \frac{V_{(p)}}{R_{(p)}}$ and the negative FDR is similar).

(9) At last, according to the definition of q-value (reference [3]), we obtain the q-value for the gene i by selecting the minimum of the FDR for the 50 delta values determined in step (7) (every delta as a reject region).

Method three, the standard different method for large samples (the amount of samples large than 80).

When the number of samples becomes large, we implement a standard analysis method for

screening difference genes. In fact, we use the two samples Welch's t-test (unequal variances) for two groups difference analysis, and use analysis of variance (ANOVA) for multiple groups (groups count no less than 3). For multiple compare analysis, we compute the q-value to control the false discovery rate (see reference [3] for details).

Step 3: the filter process for getting the significant difference gene

As the effect of different technology factors and biology factors for the experiment data, we are different to setup a unified criterion for all difference results appropriate and it has little practical significance. GCBI used an exploring way of the parameters controlled by front-end user and verified in back-end by computational process to make the result available for further analysis. The details for two groups design as follows:

The front-end user presets the q-value and fold change parameters, then we would get the difference gene count N_0 under the above setting.

(1) If N_0 is large than the threshold N_{max} of the total genes count (default is 10000 for mRNA and ncRNA, 300 for miRNA), and the total number of gene's q-value equals 0 is less than N_{max}, then we would adjust the q-value to an applicable value for filtering the analysis result. The adjusted details: we decrease q-value to the last step's q-value minus 0.01 for q-value large than 0.05, and decrease q-value to one-fifth of the last step's q-value, to make the total genes count less than N_{max}.
(2) If case (1) is violated, and the number of difference genes no less than 2, then we would output the difference result under the presetting parameters.
(3) If case (1) and case (2) are violated, and the difference gene count for presetting parameters are less than 2, we will give an error tip.
(4) If the case (1), (2) and (3) are all violated, we would select the difference result which the q-value equals to 0. Moreover, when the total gene count for genes q-value equals to 0 becomes large, we would use the strategy of increasing the d Score stepwise.

Besides, if users set the difference gene count N_{max}, then we would use the default q-value (0.75) and default fold change (1.1). Other steps are similar to the above processes for significant results.

Part two: the details of implement for clustering heatmap

For a details and pioneering application in high through-put expression profile about the heatmap, you would see reference [4] for reference. We give some details for the heatmap displayed in GCBI laboratory.

(1) Firstly, we calculate the correlation between the genes and samples. We take the Pearson correlation ρ_{ij} between gene i and gene j to construct the distance $(1-\rho_{ij})$ between them. And we implement the hierarchical clustering (used average method for linkage) based the above distance for genes and samples respectively with the expression profile.
(2) We make a median normalization for difference expression profile. It can be Represented by a mathematical formula as follows:

$$g_{ij} = g_{ij} - median(g_{i*}).$$

(3) We make a two tails truncation for the normalized values, which the low and high bound is the 10% quantile and 90% quantile respectively, and partition 20 gradual states for color hierarchy according to the truncation values. (See the key label in the top left)
(4) Moreover, we labeled the group information above the heatmap under the tree dendrogram. The samples with same group are labeled same colors. (see legend for details)

Appendix

The computation details of the Rank filed in difference result

The sort method for Fold Change Method (Method 1):

(1) For two groups design, the Rank is sorted by the absolute value of the fold change.
(2) For multiple groups design, the Rank is sorted by a weight average method for pairwise fold change (after absolute), where the weight value is (FC-10)/50+10 for FC>10, (FC-2)/8+2 for 2<FC<=10 and 0.5 for others.

The sort method for SAM (Method 2):

(1) For two groups design, the Rank is sorted by the false discovery rate q-value firstly and the absolute value of the fold change (after absolute) successively.
(2) For multiple groups design, the Rank is sorted by the positive discovery false rate q-value and d Score fields successively, where q-value is ascending and d Score is descending.

Reference:

[1] V. Tusher, R. Tibshirani, and G. Chu. Significance analysis of microarrays applied to the ionizing radiation response. Proc. Natl. Acad. Sci. USA., 98:5116–5121, 2001.
[2] G. Chu, Jun Li, B. Narasimhan, R. Tibshirani, V. Tusher. SAM "Significance Analysis of Microarrays" Users guide and technical document.
[3] J. D. Storey. A direct approach to false discovery rates. Journal of the Royal Statistical Society, Series B., 64:479–498, 2002.
[4] M. B. Eisen, P. T. Spellman, P. O. Brown, D. Botstein. Cluster analysis and display of genome-wide expression patterns. Proc. Natl. Acad. Sci. USA., 95: 14863–14868, 1998.
